# A genomic screen for angiosuppressor genes in the tumor endothelium identifies a multifaceted angiostatic role for bromodomain containing 7 (BRD7)

**DOI:** 10.1007/s10456-017-9576-3

**Published:** 2017-09-26

**Authors:** Judy R. van Beijnum, Patrycja Nowak-Sliwinska, Maaike van Berkel, Tse J. Wong, Arjan W. Griffioen

**Affiliations:** 1 Angiogenesis Laboratory, Department of Medical Oncology, Cancer Center Amsterdam, VU University Medical Center, De Boelelaan 1117, 1081 HV Amsterdam, The Netherlands; 2 School of Pharmaceutical Sciences, University of Geneva (UNIGE), Geneva, Switzerland

**Keywords:** BRD7, Angiogenesis, Tumor endothelial cells, Gene expression, CXCL1, NFκB

## Abstract

Tumor angiogenesis is characterized by deregulated gene expression in endothelial cells (EC). While studies until now have mainly focused on overexpressed genes in tumor endothelium, we here describe the identification of transcripts that are repressed in tumor endothelium and thus have potential suppressive effects on angiogenesis. We identified nineteen putative angiosuppressor genes, one of them being bromodomain containing 7 (BRD7), a gene that has been assigned tumor suppressor properties. BRD7 was studied in more detail, and we demonstrate that BRD7 expression is inversely related to EC activation. Ectopic expression of BRD7 resulted in a dramatic reduction of EC proliferation and viability. Furthermore, overexpression of BRD7 resulted in a bromodomain-dependent induction of NFκB-activity and NFκB-dependent gene expression, including ICAM1, enabling leukocyte–endothelial interactions. In silico functional annotation analysis of genome-wide expression data on BRD7 knockdown and overexpression revealed that the transcriptional signature of low BRD7 expressing cells is associated with increased angiogenesis (a.o. upregulation of angiopoietin-2, VEGF receptor-1 and neuropilin-1), cytokine activity (a.o. upregulation of CXCL1 and CXCL6), and a reduction of immune surveillance (TNF-α, NFκB, ICAM1). Thus, combining in silico and in vitro data reveals multiple pathways of angiosuppressor and anti-tumor activities of BRD7.

**Electronic supplementary material:**

The online version of this article (doi:10.1007/s10456-017-9576-3) contains supplementary material, which is available to authorized users.

## Introduction

Angiogenesis is pivotal to the progression of cancer, and inhibition of this process is considered a promising therapeutic strategy [1]. Clinical success of angiogenesis inhibition, however, is still rather limited, and problems associated with toxicity and acquired or inherent resistance to therapy are emerging. Moreover, therapeutic inhibition of the VEGF/VEGFR signaling axis was suggested to accelerate the formation of distant metastases (reviewed in [2]). We believe it is therefore necessary to gain more insight in endothelial cell (EC) biology to identify more relevant targets and design better angiostatic therapeutics.

Tumor angiogenesis is characterized by deregulated gene expression in EC, which contributes to enhanced proliferation, tube formation and matrix remodeling, to comply with the functional demands of the growing tumor. In recent years, technological improvements have facilitated the use of purified cell populations for molecular studies [3, 4], and an increasing number of putative markers of endothelium in diverse tumors have been reported [3, 5]. These markers can be targeted for therapeutic and diagnostic applications as we have demonstrated previously [3, 6, 7], and have direct clinical relevance.

In contrast to tumor cell biology, where both positive regulators (oncogenes) and negative regulators (tumor suppressor genes) have been extensively studied, focus in the field of angiogenesis has been predominantly on overexpression of genes because of targeting potential. Studies analyzing gene silencing in tumor EC are sparse and have mainly been performed in the context of promoter methylation [8–10]. In the current study, we have performed a gene expression analysis in EC of tumor- and normal tissues and queried for transcripts specifically downregulated in tumor endothelium.

We found 19 unique transcripts that were downregulated in colon tumor EC as compared to both normal colon EC and placenta EC. Of these, only very few have a reported association with cancer and/or angiogenesis. A notable exception was bromodomain containing 7 (BRD7) that triggered our interest by its previously proposed tumor suppressor function in different types of cancer [11–14].

Bromodomain proteins are a large family of chromatin binding molecules that are highly conserved through evolution. BRD7 is a subunit of the PBAF-specific SWI/SNF chromatin remodeling complex [15, 16]. It binds to acetylated histones and serves to modulate transcription factor activity [15, 17]. Recently, it was shown that BRD7 binds directly to p53 and is required for efficient transcription of a subset of p53 target genes, such as p21 and PAI-1 [11, 18]. In addition, direct binding of BRD7 to BRCA1 was demonstrated [15]. Furthermore, XAF1, another transcriptional target of BRD7, is involved in endothelial cell senescence [19].

In this study, we investigated the role of BRD7 in tumor angiogenesis. We demonstrate an inverse relation between BRD7 expression levels and activation status of EC, both in vitro and in vivo. We show that suppression of BRD7 induces a pro-angiogenic cytokine and gene expression profile, whereas increased expression of BRD7 induces NFκB-mediated ICAM1 expression which promotes anti-tumor immunity [20, 21]. Thus, interference in BRD7 expression offers therapeutic potential for inhibition of angiogenesis and tumor growth via multiple mechanisms.

## Materials and methods

### Isolation and culture of endothelial cells

**Fig. 1:**
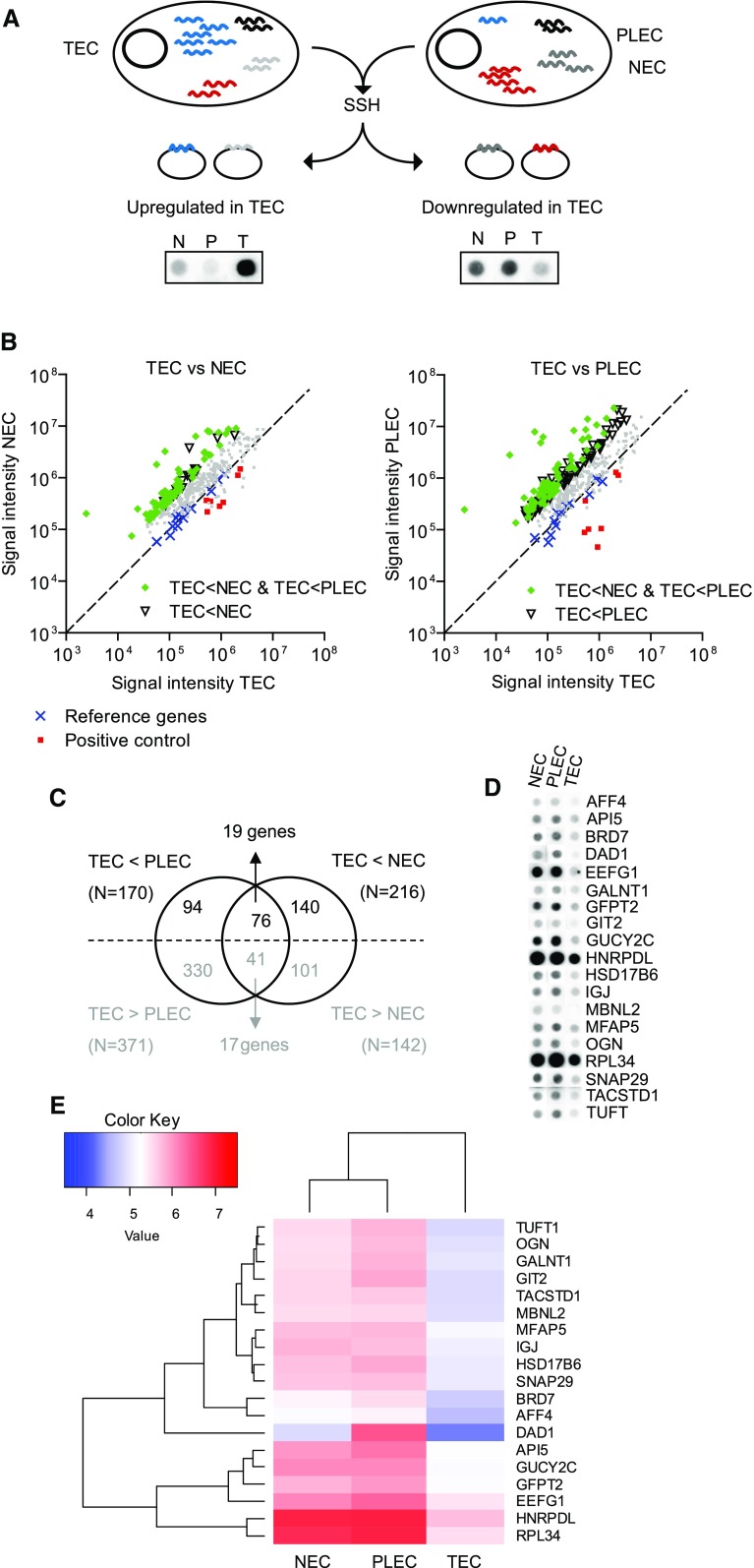
Identification of genes downregulated in tumor EC. **a** Schematic overview of suppression subtractive hybridization (SSH) and differential (cDNA array) screening. Transcripts differing in abundance between tumor EC (TEC) and placenta EC (PLEC) or normal EC (NEC) are cloned in cDNA libraries. **b** The subtracted and enriched cloned repertoires were arrayed and probed with cDNA from TEC, NEC and PLEC. Shown are spot intensities in comparisons between TEC and NEC (left panel) and between TEC and PLEC (right panel). A clear bias toward genes suppressed in TEC is apparent, confirming successful subtraction. Spots showing > 3.5-fold expression differences in either comparison (triangles) or in both comparisons (green diamonds) are indicated, as are the reference genes (blue crosses) and positive controls (red squares). **c**, **d** 170 spots showed > 3.5-fold expression difference in TEC versus NEC and 216 spots showed > 3.5-fold lower expression in TEC versus PLEC. 76 clones showed overlapping profiles (**c**) and were shown to represent 19 different mRNA transcripts (**d**). **e** Heatmap and unsupervised clustering on average Log10 normalized intensities (see Table S1) of the top gene list

EC were isolated from colorectal tumor tissues, patient-matched normal colon tissues and placenta tissues as previously described [3, 4]. Custom cDNA array screening [3] is detailed in the supplementary material and Fig. 1. HUVEC, HMEC-1 (referred to as HMEC, [22]) and EC-RF24 (referred to as RF24, [23]) were cultured as previously described [24, 25]. Where indicated, cells were treated for 3 days with 2 µM sunitinib (Pfizer).

### Developmental CAM

The CAMs on embryonic development day (EDD) 7 were divided into two treatment groups: control (topically 0.1% DMSO in 0.9% NaCl; 100 μl injected) and sunitinib (topically 3.2 μg/embryo/day). The same treatment was repeated on EDD8. The CAMs were excised on EDD9 and processed for RNA isolation.

### Human tumors grown on the CAM

Human ovarian carcinoma (A2780), human colorectal carcinoma (HCT116) and human breast carcinoma (MDA-MB-231) (ECACC, Salisbury, UK) were maintained at 37 °C and 5% CO_2_ in RPMI-1640 cell culture medium, supplemented with 10% FCS. A2780 and MDA-MB-231 tumors were implanted onto the CAM on EDD8 using hanging drops by preparing 25-μl drops containing 1 × 10^6^ cells, whereas for HCT116 2.5 × 10^6^ cells were applied in a 1:1 mix of medium and Matrigel (BD Biosciences). Where indicated, vascularized A2780 tumors were measured at EDD10, randomized and treated with vehicle (i.v. 0.1% DMSO in 0.9% NaCl; 100 μl injected) or sunitinib (i.v. 12 μg/embryo/day in 100 μl vehicle). Xenograft tumors were excised on EDD15 and processed for RNA isolation.

### Quantitative real-time reverse transcription-PCR

Total RNA was isolated using the RNeasy RNA isolation kit (Qiagen) and used for cDNA synthesis (Bio-Rad) according to the supplier’s protocols. Cells were harvested by trypsinization and pelleted prior to cell lysis. Freshly resected colon tumor and adjacent normal colon tissue were minced and processed for RNA isolation.

Primer design and validation was performed as described previously [26]. Primers specific for chicken BRD7, to selectively measure vascular BRD7 expression in the xenografted tumors, are presented in Fig. S1, and additional sequences are listed in Table S2. qPCR was performed using SYBR Green reagent (Bio-Rad). Assays were run on a CFX96 Real-Time PCR detection system and analyzed using Bio-Rad CFX Manager software. Expression levels were defined using the 2^(−dCt) using mean expression of cyclophilin A (PPIA), beta-actin (ACTB) and beta-2-microglobulin (B2M) as reference genes. Where relevant, expression levels were expressed relative to one condition under study.

### BRD7 overexpression and knockdown

Constructs containing Flag-tagged full-length (BRD7-FL) and bromodomain-deleted (aa129-237; BRD7-dBr) BRD7 open reading frames in pcDNA3 were a kind gift of Dr. Julia Kzyshkowska [27], and similar constructs in pEGFPN1 a kind gift of Dr. Jarno Drost [11]. For transfection, 1 × 10^5^ RF24 cells were combined with 500 ng pDNA in resuspension buffer R (Life Technologies) in a 10-µl tip and subject to 3 pulses of 10 ms at 1600 V in the Neon ^®^ Transfection system (Life Technologies). After electroporation, cells were seeded in 2 wells of 24-well plates to recover and used in downstream experiments after 48 h. Controls represent cells transfected with the corresponding empty vectors.

Three different siRNAs targeting BRD7 were obtained from Qiagen (FlexiTube; SI04134088, SI04168059 and SI04320204), and control scrambled siRNA was obtained from Eurogentec. HUVEC were reverse transfected with 50 nM siRNA as previously described [6].

### Functional assays

EC proliferation was measured using a ^3^H-thymidine incorporation assay as described previously [3, 24]. For chemotaxis measurements, near-confluent HUVEC were starved overnight in medium containing 0.1% FCS prior to seeding 2.5 × 10^4^ cells in a FluoroBlok insert with 8 µm pore size (BD Biosciences) in a 24-well plate. The lower compartment of the well was filled with 200 µl medium (0.1% FCS) combined with 50 µl conditioned medium of siRNA-transfected HUVEC. Plates were incubated overnight, and cells on the bottom side of the filter were stained with Calcein AM (Life Technologies). Five areas of the filters were photographed, and migrated cells were counted.

Active p65 NFκB subunit was detected using the EZ-detect NFκB p65 ELISA assay kit (Pierce), according to the manufacturer’s instructions. Briefly, cell lysates were incubated with p65 target DNA sequence immobilized in the assay plate. DNA-bound p65 was detected using antibodies in combination with chemiluminescence. Values were normalized to total protein content.

### Data mining and bioinformatics analysis

Publicly available repositories were queried to obtain additional information on BRD7 gene and protein expression. The Protein Atlas (http://www.proteinatlas.org/) staining data were used to compare BRD7 protein expression in healthy and cancer tissues. NCBI GEO datasets GSE20076 [11] and GSE22607 [18] were analyzed to unravel the effects of BRD7 knockdown. GSE22607 contains expression data on BJ1 fibroblasts transduced with shBRD7 and GSE20076 contains expression data on RasV12 expressing BJ1 fibroblasts transduced with shBRD7. Briefly, using the GEO2R functionality, normalized log2FC values of shBRD7-treated samples versus control samples were obtained and read into R. In addition, the subsets of genes showing differential expression after transfection of BRD7 in HEK293 fibroblasts reported by Xu et al. [28] were added as the full data set (GSE53656) was not publicly accessible. Data sets were merged and subsequently aggregated on gene names to have one unique identifier representing average expression of low versus high BRD7 expressing cells. FunRich (www.funrich.org), DAVID (https://david.ncifcrf.gov/), WebGestalt (www.webgestalt.org) and PathVisio (www.pathvisio.org) were used to perform functional annotation analysis on the final data set.

### Statistical analyses

All values are given as mean values ± SEM. Statistical analyses were done using either *t* test, Mann–Whitney *U* (M–W) or Wilcoxon rank sum test (Wilcoxon) for single comparisons, or, where appropriate, one-way ANOVA or Kruskal–Wallis (K–W) in combination with Dunnett’s multiple test correction. All analyses were done in GraphPad Prism 3.0. *p* values < 0.05 were considered statistically significant.

## Results

### BRD7 expression is inhibited in tumor endothelium

Gene expression profiling of freshly isolated endothelial cells (EC) from colon tumors, normal colon and placenta identified 19 genes that were specifically suppressed in tumor EC (TEC) (Fig. 1; Table S1). The reported downregulation of BRD7 in cancer [13, 14, 29] prompted us to further elucidate the role of BRD7 in tumor angiogenesis.

**Fig. 2:**
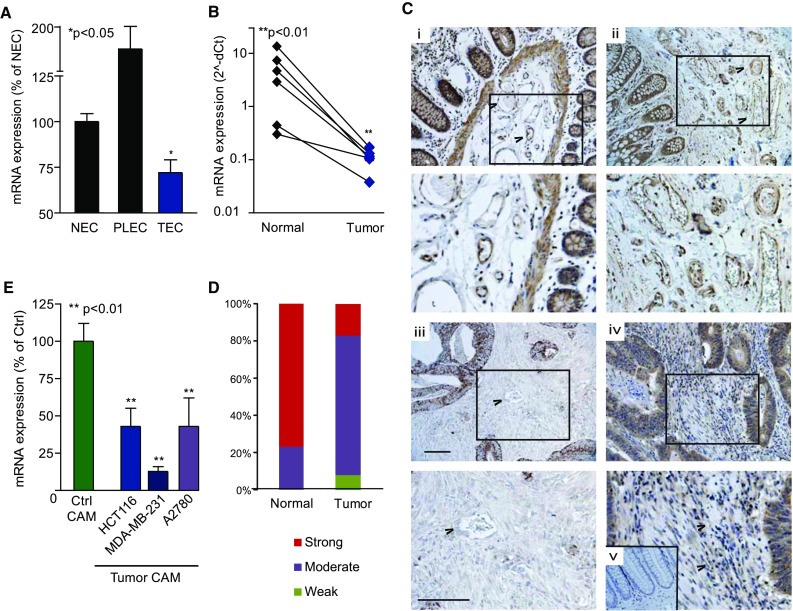
Expression of BRD7 is suppressed in tumor vasculature. **a** BRD7 expression is substantially reduced in tumor EC (TEC) as compared to normal EC (NEC) and placenta EC (PLEC) as shown by qPCR. *p < 0.05 Kruskal–Wallis test. **b** qPCR analysis of total tumor RNA confirms the global suppression of BRD7 in tumors. **p < 0.01 paired *t* test. **c** BRD7 protein is detected in EC and underlying structures (e.g., muscular layers) of blood vessels as well as in the crypts of normal colon tissue (*i*, *ii*). Expression of BRD7 is markedly reduced in colon tumors, especially in the vasculature, and in general the expression is much more diffuse (*iii*, *iv*). Negative control staining is shown in panel *v*. Boxed areas are magnified in their respective panels below. For indicative purposes, several blood vessels are indicated with arrowheads. Scale bar = 100 µm. **d** Distribution of staining intensities in colon tumors and normal colon samples (*N* = 12) reported in The Protein Atlas indicates less intense staining in tumor tissues. **e** BRD7 mRNA expression is reduced in the (chicken derived) vasculature of different human tumors (colon, breast and ovary) xenografted on the CAM (Tumor CAM) in comparison with developmental CAM (Ctrl CAM), as determined by qPCR using chicken-specific primers. **p < 0.01 ANOVA. All data are presented as mean ± SEM

qPCR validated the differential BRD7 expression in isolated EC. Not only is BRD7 mRNA specifically downregulated in TEC (Fig. 2a), global BRD7 mRNA expression was reduced in a panel of colorectal tumors compared to normal colon (Fig. 2b), confirming previous reports [30]. BRD7 protein in normal colon tissue sections was clearly associated with the vasculature (Fig. 2c i, ii), both in the endothelial cell layer and in underlying vascular structures such as the vascular smooth muscle layer. Vascular BRD7 expression was virtually absent in colon tumor sections (Fig. 2c iii, iv). In addition, mining The Protein Atlas data also revealed a reduction in BRD7 protein expression in colon tumors (Fig. 2d) as compared to normal colon.

While in tumor cells loss of chromosome 16q12 may be responsible for reduced expression of BRD7 [31], loss of heterozygosity is not a common phenomenon in TEC which are considered non-transformed cells [32]. Epigenetic silencing of BRD7 expression through promoter CpG island methylation and/or histone deacetylation in EC would be feasible [9, 33], but was not observed (Fig. S2).

Suppression of tumor EC BRD7 expression appears to be a generic feature in cancer. Using PCR primers specific for chicken BRD7 (Fig. S1), we profiled the vascular BRD7 expression in different human tumors (colon, breast and ovary) xenografted on the chicken chorioallantoic membrane (CAM) [34–36]. Figure 2e clearly shows suppressed BRD7 expression in the tumor blood vessels on the CAM as compared to normal CAM vessels (Ctrl CAM). Thus, in addition to its tumor suppressive properties, the expression profile of BRD7 is also suggestive of angiosuppressor functions.

### BRD7 expression is negatively associated with endothelial cell activation

**Fig. 3:**
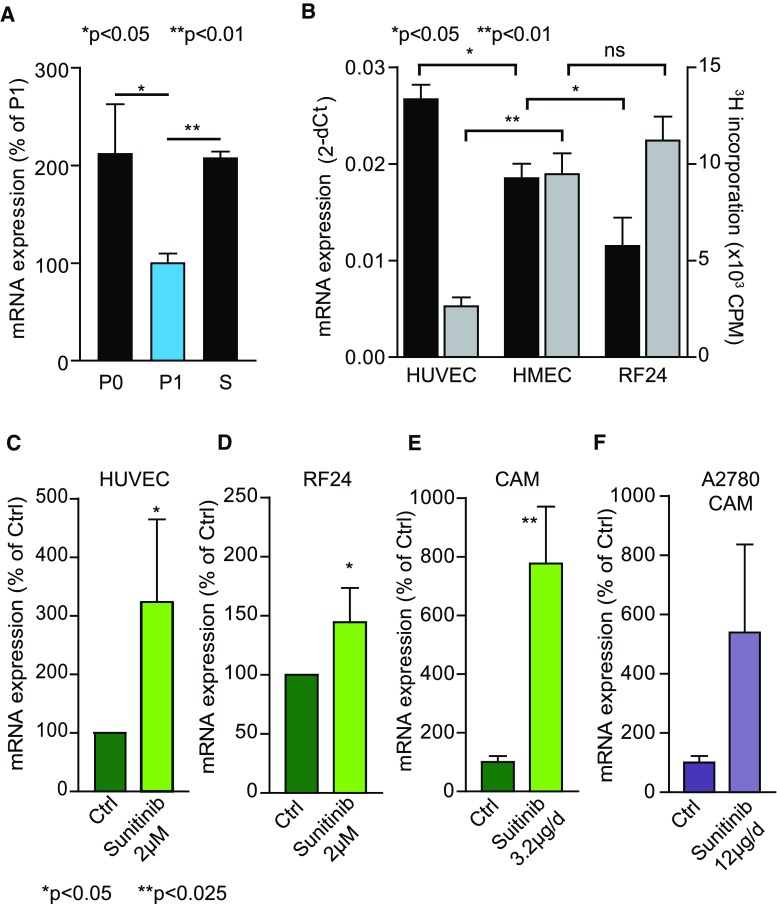
BRD7 expression is inversely related to endothelial cell growth. **a** HUVEC were isolated as described and either processed for RNA isolation immediately after isolation (P0) or grown under standard conditions for 3 days (P1), and BRD7 transcript levels were measured by qPCR. A clear reduction of BRD7 mRNA is evident in cells stimulated to proliferate in vitro. Serum withdrawal (S) of established HUVEC increases the BRD7 mRNA levels to that of primary isolates. *p < 0.05, **p < 0.01 *t* test. **b** BRD7 expression was measured in routinely cultured HUVEC, HMEC and RF24 by qPCR. In parallel, proliferation rate of the cells was measured by ^3^H-thymidine incorporation. Primary cells (HUVEC) show higher expression levels (black bars; left *y*-axis) than immortalized EC (RF24 and HMEC), but the opposite holds true for proliferation rate (gray bars; right *y*-axis). *p < 0.05 Wilcoxon test. **c**, **d** HUVEC (**c**) and RF24 (**d**) were treated with 2 µM sunitinib and BRD7 was quantified by qPCR. Sunitinib markedly increases BRD7 expression. *p < 0.05 Wilcoxon test. **e** CAMs were treated with 3.2 µg sunitinib per day, and chicken BRD7 expression was determined with qPCR. Similar to in vitro treatment, in vivo treatment with sunitinib increases BRD7 expression. **p < 0.01 ANOVA. **f** sunitinib treatment (12 µg/day) of A2780 tumors on the CAM results in an increase in vascular BRD7 expression as determined by qPCR. *p = 0.05 Mann–Whitney test. All data are presented as mean ± SEM

To determine how BRD7 expression is regulated in EC, freshly isolated quiescent human umbilical vein EC (HUVEC) (P0) were compared with cultured, serum-activated HUVEC (P1), demonstrating a clear suppression of BRD7 transcript upon propagation in vitro (Fig. 3a). Restoration of original BRD7 expression levels was accomplished by serum withdrawal (S) (Fig. 3a). Serum depletion of HMEC and RF24 also resulted in enhanced BRD7 expression, comparable to that seen for HUVEC (Fig. S3). In addition, when the expression levels of BRD7 in the immortalized, fast-growing EC lines HMEC and RF24 were compared with that of primary HUVEC, a markedly higher expression was observed in primary, slower-growing HUVEC, further suggesting an inverse association between BRD7 transcript levels and EC proliferation rates (Fig. 3b). Thus, expression of BRD7 appears to be influenced by microenvironmental stimuli, as well as by endogenous growth rates of the EC.

The clear relationship between BRD7 expression levels and EC growth prompted us to investigate whether BRD7 expression is altered upon treatment of EC with angiogenesis inhibitors. EC were treated with sunitinib, and BRD7 expression was monitored by qPCR. The receptor tyrosine kinase inhibitor sunitinib, but not the anti-VEGF antibody bevacizumab (data not shown), induced the expression of BRD7 (Fig. 3c, d), which corresponds to the effective inhibition of EC growth in vitro by sunitinib though not bevacizumab [36, 37]. A similar expression regulation of BRD7 was observed in the developmental CAM in vivo (Fig. 2e). Xenograft tumors treated with sunitinib showed enhanced expression of endothelial BRD7 (Fig. 3f) and displayed inhibited growth [35]. Together, these data further support the notion that BRD7 expression levels are negatively associated with EC growth.

### BRD7 exerts an angiosuppressive function

**Fig. 4:**
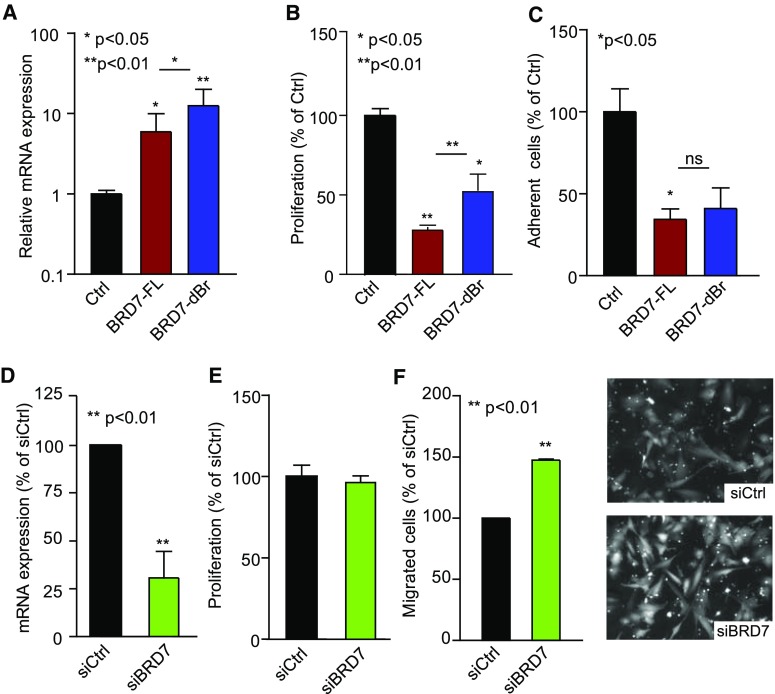
Functional effects of BRD7 overexpression and knockdown in endothelial cells. **a**, **b** Full-length BRD7 (BRD7-FL) and bromodomain deletion mutant (BRD7-dBr) cloned in pcDNA3 [27] were ectopically expressed in RF24. Control cells were transfected with empty pcDNA3. This resulted in seven to tenfold increased in BRD7 expression (**a**). BRD7 overexpression results in inhibition of EC proliferation as measured by ^3^H-thymidine incorporation assay (**b**). **c** Overexpression of BRD7 and BRD7-dBr results in a reduced fraction of adherent cells. Cells were transfected with GFP-tagged BRD7 expression constructs or empty pEGFPN1 vector, which allowed easy identification of successfully transfected cells. *p < 0.05, **p < 0.01 ANOVA. **d**–**f** Knockdown of BRD7 by siRNA in HUVEC (**d**) does not affect proliferation of cells (**e**). Migration toward conditioned medium of siBRD7-treated cells was enhanced (**f**). **p < 0.01 *t* test. All data are presented as mean ± SEM

To functionally evaluate the role of BRD7 and specifically its bromodomain in EC biology, RF24 cells were transfected with previously validated expression constructs encoding either full-length BRD7 protein (BRD7-FL) or BRD7 protein with deleted bromodomain (BRD7-dBr) [11, 27], resulting in a clear induction of BRD7 expression (Fig. 4a). Transfection efficiencies were at least 50%, comparable for the different constructs, consequent for mRNA and protein induction, and displayed only moderate impairment of cell viability. In addition, we observed no difference in expression of BRD7 in empty vector-transfected cells as compared to untransfected cells, essentially ruling out artifacts related to the procedure (Fig S4 and data not shown). We next investigated the cell growth properties of EC transfected with the different BRD7 expression constructs. As is evident from Fig. 4b, introduction of BRD7 resulted in a drastic inhibition of EC proliferation, which was partially overcome by deletion of the bromodomain. Comparable results were obtained with GFP-tagged and Flag-tagged BRD7 expression constructs. Moreover, using GFP-tagged expression constructs, which allowed direct monitoring of successfully transfected cells, we observed that the majority BRD7 expressing GFP-positive cells failed to adhere to the growth substrate indicating severe loss of viability and adhesive properties upon BRD7 expression (Fig. 4c). Since five to tenfold overexpression resulted in considerable suppression of EC proliferation, we considered these cells too impaired for further assessment of sprouting capacity.

Using siRNA to knock down BRD7 expression, we sought to reverse the phenotypic effects observed with the expression constructs. BRD7 expression was profoundly suppressed (Fig. 4d). However, we did not observe effects on EC proliferation (Figs. 4e, S4) and scratch wound migration (data not shown). Comparable results were obtained with two of the three independent BRD7-specific siRNAs (Fig S4 and data not shown). All data were expressed relative to a scrambled siRNA control as to exclude off-target effects.

The lack of phenotype may be related to the intrinsically high activation status of cultured EC in vitro, which leaves a too narrow detection window for additional activation as a consequence of BRD7 suppression. Of note, we selected HUVEC for these experiments as they express the highest levels of BRD7 and display the lowest level of proliferation when compared to HMEC and RF24 (Fig. 3b). Furthermore, serum starvation of the cells after the transfection procedure did not induce any divergent responses in siBRD7- versus siCtrl-transfected cells. However, chemotactic migration of naïve cells toward conditioned medium of siBRD7-treated cells was enhanced (Fig. 4f) and appeared to be associated with more intense Calcein AM fluorescence (Fig. 4f, right panel), suggestive of increased viability. Nevertheless, quantification of fluorescence intensity did not reveal a significant increase (data not shown).

### BRD7 affects inflammatory and angiogenic cytokine expression

**Fig. 5:**
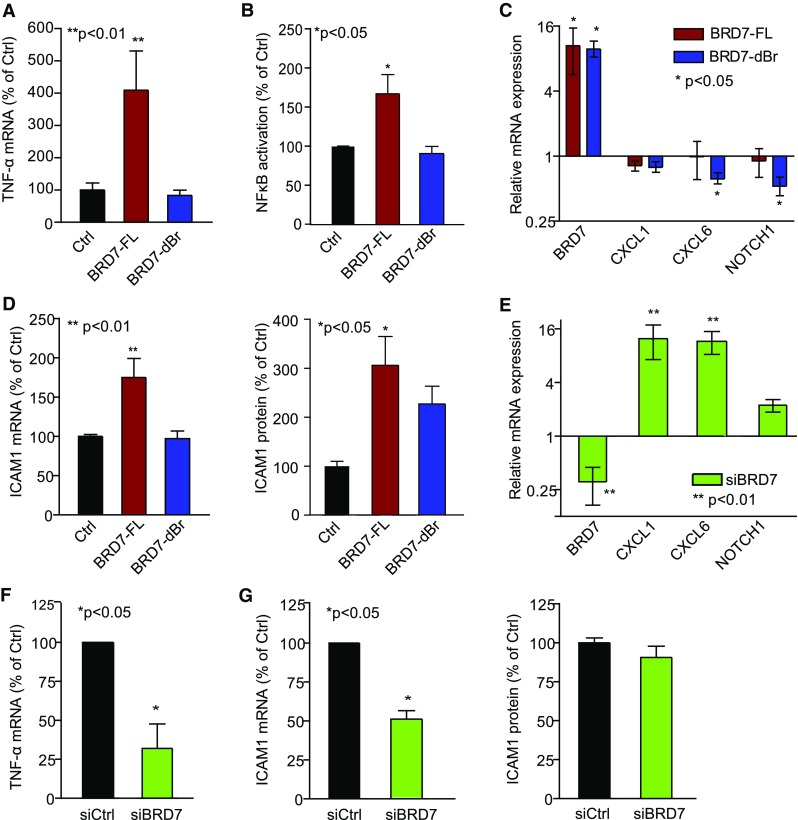
BRD7 expression modulation affects angiogenic and inflammatory gene expression. **a**–**d** Full-length BRD7 (BRD7-FL) and bromodomain deletion mutant (BRD7-dBr) were ectopically expressed in RF24, which resulted in a bromodomain-dependent increase in TNF-α expression (**a**), NFkB activation (**b**), and ICAM1 mRNA and protein (**d**). Expression of CXCL1, CXCL6 and NOTCH1 is only slightly affected (**c**). **e**–**g** mRNA expression changes are reversed upon knockdown of BRD7, although ICAM1 protein expression was not affected. *p < 0.05, **p < 0.01 ANOVA. All data are presented as mean ± SEM

To further elucidate the mechanism by which BRD7 affects EC activation, we profiled a panel of angiogenic factors and their receptors in BRD7-transfected (BRD7-FL and BRD7-dBr) or empty vector-transfected EC (Ctrl) by qPCR. From Fig. S4a, it is clear that overexpression of BRD7-FL or BRD7-dBr does not have a major influence on the expression of angiogenic growth factors and their receptors involved in signaling along the VEGF/VEGFR or angiopoietin/Tie axis. Moderate changes were observed with BRD7 knockdown, most notable the upregulation of VEGF receptor-1 (FLT1), angiopoietin-2 (ANGPT2) and neuropilin-1 (NRP1) (Fig. S4b, c). In contrast, TNF-α was markedly upregulated after overexpression of full-length BRD7 but not after overexpression of the bromodomain-deficient protein (Fig. 5a), and this was paralleled by an increase in the TNF-α target ICAM1 mRNA and protein (Fig. 5d) [8, 9]. In agreement with this, TNF-α and ICAM1 mRNA expression were repressed by knockdown of BRD7 (Fig. 5f, g), although protein levels were seemingly unaffected (Fig. 5g). As ICAM1 is a downstream target of TNF-α signaling via NFκB [38], we set out to investigate NFκB activation status in EC transfected with BDR7-FL and BRD7-dBr. Figure 5b demonstrates that ectopic expression of BRD7-FL, but not BRD7-dBr, induces the activity of NFκB subunit p65 (RelA).

**Fig. 6:**
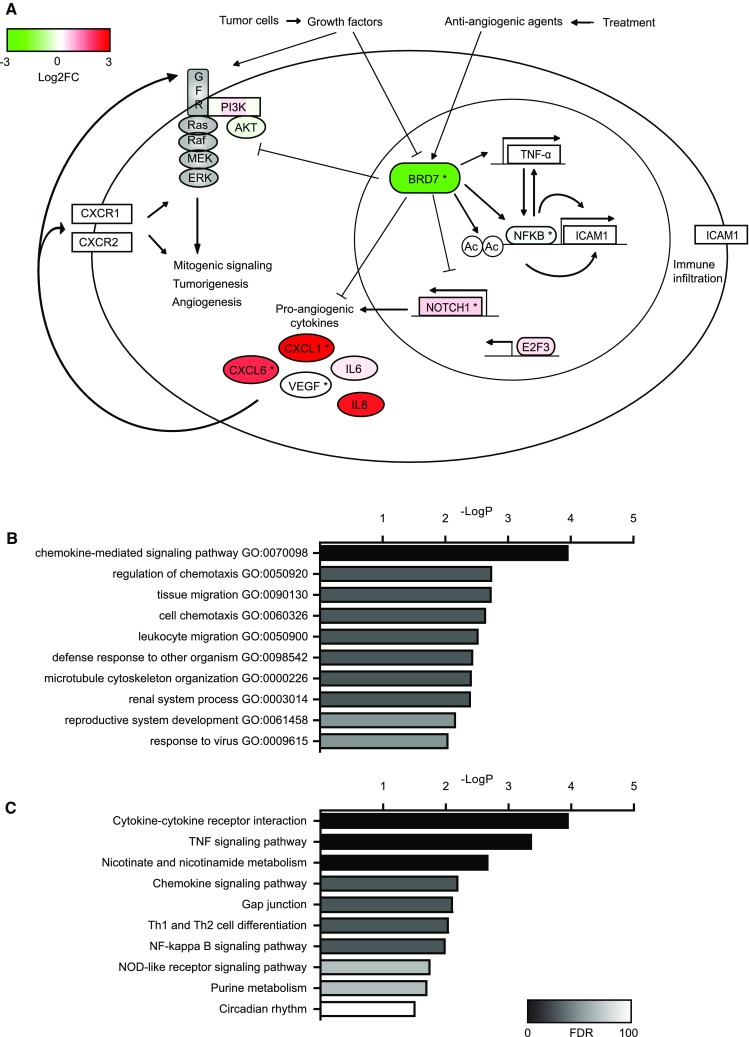
Schematic representation of the multifaceted angiosuppressor function of BRD7. **a** BRD7 expression is stimulated by angiogenesis inhibitors and repressed by growth factor exposure, such as those produced by tumor cells. BRD7 inhibits mitogenic Ras-Raf-MEK-ERK and PI3K-Akt signaling, as well as E2F3 transcription factor activity and NOTCH1 expression that also normally result in enhanced cell growth and contribute to angiogenesis and tumorigenesis. Low BRD7 levels are related to enhanced CXCL1, CXCL6, IL6 and IL8 cytokine production, which can also promote tumor growth and angiogenesis. On the other hand, BRD7 can directly or indirectly increase TNF-α expression and stimulates NFκB activation, resulting in increased expression of downstream target genes including ICAM1. Furthermore, BRD7 might also directly facilitate ICAM1 transcription by interaction with acetylated histones in the ICAM1 promoter sequence. Upregulation of ICAM1 results in increased EC–leukocyte interactions and positively contributes to immune cell infiltration into tumors. Colors reflect average expression changes in the analyzed data sets. * indicates concordant regulation in endothelial cells reported in this work. **b**, **c** Overrepresentation analysis of gene ontologies (B) or KEGG pathways (**c**) in differentially expressed (Log2FC > 2) genes (*N* = 290) after BRD7 knockdown using WebGestalt tool. Top 10 categories are shown with *p* values. Bars are color-coded for false discovery rate (FDR). Of note, no significantly enriched ontologies were found with Log2FC < 2 genes following BRD7 knockdown (*N* = 360) (data not shown)

To further elucidate potential mechanisms of angiosuppressive actions of BRD7, we employed analysis of publicly available gene expression data sets on BRD7 overexpression and knockdown [11, 18, 28]. Comparison of the individual data sets revealed that only limited overlap of concordantly regulated genes was apparent (Fig. S5, Table S3). Functional annotation clustering analysis of the different data sets on gene ontologies using DAVID, however, showed several similarities. The fraction of genes associated with low BRD7 expression displayed enrichment for genes associated with inflammatory responses and cytokine activity, as well as for angiogenesis (Table S5). A similar profile was apparent for ontology analysis of the merged data set (Fig. 6b, c).

Cytokines CXCL1 and CXCL6 were differentially regulated in all three expression datasets under investigation and were confirmed to be upregulated after BRD7 knockdown and somewhat suppressed after overexpression EC (Fig. 5c, e). In addition, NOTCH1, previously identified as a transcriptional target of BRD7 [28], was subject to BRD7-induced regulation (Fig. 5c, e).

Using PathVisio [39] and the merged expression data set containing average Log2FC values, we constructed a schematic representation on BRD7 angiosuppressive actions with expression changes color-coded (Fig. 6a). Downregulation of BRD7 was accompanied by NOTCH1, CXCL1 and CXCL6 upregulation and NFκB downregulation, which we experimentally confirmed (Fig. 5). Using overrepresentation analysis, pathways and ontologies associated with cytokine signaling, chemotaxis and migration, as well as NFκB and TNF-α signaling were shown to be enriched in cells with suppressed BRD7 expression (Fig. 6b, c). Together, these data point to a multifaceted action of BRD7 in regulating the expression of different angiogenic mediators.

## Discussion

In this report, we describe the identification of BRD7 as a negative regulator of angiogenesis. BRD7 expression was found to be suppressed in endothelial cells (EC) isolated from human colon tumors as compared to isolated EC from normal colon tissue. BRD7 has been described as a putative tumor suppressor protein, which prompted us to further investigate its role in tumor angiogenesis.

We confirmed suppressed BRD7 expression both in isolated EC and reduction of BRD7 expression in tumor endothelium was independently observed in different xenografted tumors on the CAM, suggesting a generic cancer feature. Unfortunately, no relevant public data sets to independently validate BRD7 expression in human isolated TEC are available. The sparse availability of tissue-isolated EC precluded us also from quantifying BRD7 protein levels in these cells separately. However, immunohistochemistry of colorectal cancer and normal tissues confirms the downregulation of BRD7 protein in the tumor vasculature. In concordance with these findings, BRD7 expression levels were inversely related with EC activation status in vitro. In addition, treatment with the anti-angiogenic tyrosine kinase inhibitor (TKI) sunitinib increased BRD7 expression levels in vitro and in vivo. This relatively broad spectrum TKI may affect multiple phosphorylation pathways that (in)directly affect the regulation of BRD7 expression.

Ectopic expression of BRD7 resulted in decreased proliferation of EC, and this phenotype was partially dependent on the presence of the bromodomain. So far, deletion of the bromodomain in BRD7 has been associated with a reduced binding capacity to acetylated histones [27], and a loss of inhibition of E2F3 promoter activity [17, 29]. Also, nuclear expression of BRD7 lacking bromodomain is more granular [17], likely caused by an altered ability to bind chromatin. However, the precise mode of action of BRD7, and more specifically the role of its bromodomain in regulating cell growth has not been fully elucidated. Our data indicate that the bromodomain of BRD7 is involved in regulating NFκB activation and the expression of downstream molecules (TNF-α, ICAM1), but not that of other molecules such as CXCL1, CXCL6 and NOTCH1.

In previous studies [8, 9], we profiled epigenetic silencing in endothelial cells and demonstrated a pivotal role for ICAM1. Endothelial ICAM1 is one of the key adhesion molecules that mediate leukocyte rolling and adhesion, and hence contributes to leukocyte infiltration and anti-tumor immunity. In tumors, however, the expression of ICAM1 on EC is suppressed, and thus, tumors may escape from immune surveillance [8, 20, 21]. As we have demonstrated in the past, anti-angiogenic treatment can overcome the downregulation of ICAM1 and restore leukocyte infiltration [20], and as shown here, this is accompanied by, and may be a direct consequence of, an increase in BRD7 expression. ICAM1 can be regulated via TNF-α-induced NFκB-dependent transcription, but TNF-α expression itself can also be controlled by NFκB, creating a positive feedback loop [21]. Binding of NFκB p65 subunit to its target sequence was enhanced in cells overexpressing BRD7-FL, but not BRD7-dBr, suggesting BRD7 stimulates NFκB-dependent transcription in a bromodomain-dependent fashion. Though we did not investigate direct binding of BRD7 to NFκB, in this context, it is highly interesting to note that recently the related BRD4 protein was shown to bind to RelA and to activate NFκB by a bromodomain-dependent mechanism [40]. Alternatively, BRD7 could, through its bromodomain, bind acetylated histones that are present in the ICAM1 promoter and positively regulate its expression [8]. A recent chromatin-IP study did, however, not identify these genes as direct transcriptional targets of BRD7 [28]. Nevertheless, BRD7 is known to act in larger complexes and interactions may be cell-type dependent [11, 15, 18].

NFκB plays a paradoxical role in tumor growth and tumor angiogenesis. In tumor cells, its activation is altered and it drives the production of pro-angiogenic cytokines, including CXCL1 and CXCL6. In addition, it feeds a positive pro-metastatic and pro-angiogenic feedback loop [41] and inhibits pro-apoptotic genes [38]. However, in EC NFκB plays a divergent role, as it may play a predominant pro-apoptotic role, induces the expression of anti-angiogenic factors and is involved in effectuating angiostatic therapy and increasing anti-tumor immunity [38]. In addition, in HUVEC, but not in fibroblasts, it plays a pivotal role in senescence induction [42, 43], whereas blockade of NFκB could overcome aging [44]. Interestingly, recently XAF1, a transcriptional target of BRD7 which acts analogously to NFκB in inhibiting XIAP, was shown to play a role in endothelial senescence [19].

The data presented in this study also point to a unique positioning of NFκB in EC. While studies in fibroblasts employing BRD7 expression modulation report induction of the pro-angiogenic cytokines CXCL1 and CXCL6 upon BRD7 silencing, upregulation of ICAM1 and NFκB are concomitantly observed [11, 18, 28, 45]. In contrast, in EC BRD7 acts in an opposite manner on NFκB and ICAM1. To further unravel the differential actions of BRD7 in relation to NFκB-mediated processes, as well as its in vivo contribution of tumor angiogenesis, the generation of genetically engineered models of (conditional) vascular BRD7 knockout, combined with ChIP-seq analysis, would be highly valuable.

BRD7 has been described as a subunit of the SWI/SNF chromatin remodeling complex PBAF [16]. Involvement of this complex in angiogenesis was shown by knockdown of the accompanying BRG1 subunit in embryonic stem cells, which resulted in the induction of angiogenesis associated genes [16]. However, a later study employing vascular specific knockout of BRG1 yielded no observable effects on postnatal angiogenesis [46]. While BRG1 can participate in both BAF and PBAF, BRD7 is specific for PBAF [16]. As such, depletion of BRG1 implies a functional deletion of BRD7 action within this complex. Indeed, transcriptional changes upon BRG1 knockdown mimicked those of BRD7 suppression such as shown here [16, 47, 48]. The apparent absence of phenotype upon knockdown is in line with our observations that suppression of BRD7 expression has less effects than overexpression, suggesting that compensatory mechanisms likely play a role.

Endothelial BRD7 contributes to suppression of tumor angiogenesis in a number of ways, schematically visualized in Fig. 6 and reconstructed from data presented here combined with published data. BRD7 inhibits mitogenic signaling, tumorigenesis and angiogenesis and stimulates anti-tumor immunity through different possible pathways. BRD7 negatively regulates the Ras-Raf-MEK-ERK mitogenic and PI3K-Akt signaling cascades [17, 28, 29, 49], the latter presumably through direct binding of PI3K subunit p85α [49]. Furthermore, BRD7 inhibits E2F3 [17, 29, 50] and NOTCH1 [28] promoter activity. Interestingly, blockade of the NOTCH signaling pathway has been employed to target tumor angiogenesis, although the complex interplay of its different ligands complicate predicting the outcome of such strategies [51]. The discovery that knockdown of BRD7 results in induction of pro-angiogenic cytokines, most concordantly CXCL1 and CXCL6 expression (Figs. 5, 6, S5; Tables S3, S4), adds an additional layer to its angiomodulatory actions. CXCL1, previously known as GRO1 oncogene, has mitogenic, angiogenic and tumorigenic properties [52]. CXCL6, also known as GCP-2, is another pro-angiogenic cytokine with tumor growth promoting properties [53], and anti-CXCL6 antibodies reduced tumor growth and metastasis formation in vivo [54]. Moreover, CXCL6 can stimulate the production of other pro-angiogenic factors such as VEGFA, HGF and IL8 [55]. Interestingly, the previously reported tumor angiogenesis gene HMGB1 [3, 6, 56] displays parallel actions to BRD7 knockdown as it induces the expression of different pro-angiogenic cytokines, including IL8, CXCL1 and CXCL6 [57]. This might feed an autocrine and/or paracrine positive feedback mechanism [56], where tumor cells and EC can reciprocally stimulate each other’s growth [58], contributing to tumor progression and sustained angiogenic activation [17, 28, 29, 49].

In summary, we identified a novel angiosuppressor gene that extends our understanding of the physiology of tumor EC and provides multiple novel opportunities for interfering with tumor angiogenesis. Therapeutic induction of BRD7 expression might not only be valuable in reducing tumor cell proliferation, but also in reducing tumor angiogenesis and increasing anti-tumor immune infiltration. This proposed triple-targeting of cancer progression offers a unique and new approach in the battle against cancer, and may prove superior to monotherapies.

## Electronic supplementary material

Below is the link to the electronic supplementary material.

Supplementary material 1 (DOCX 36 kb)

Supplementary material 2 (PDF 84 kb)

Supplementary material 3 (PDF 61 kb)

Supplementary material 4 (PDF 70 kb)

Supplementary material 5 (PDF 56 kb)

Supplementary material 6 (PDF 55 kb)

Supplementary material 7 (XLSX 28 kb)

Supplementary material 8 (XLSX 939 kb)

## Abbreviations

EC: Endothelial cell
BRD7: Bromodomain containing 7
TEC: Tumor EC
NEC: Normal EC
PLEC: Placenta EC
HUVEC: Human umbilical vein endothelial cell
qPCR: Quantitative real-time PCR
CAM: Chicken chorioallantoic membrane
VEGF: Vascular endothelial growth factor
TKI: Tyrosine kinase inhibitor
TNF-α: Tumor necrosis factor-α
NFκB: Nuclear factor kappa B
ICAM1: Intercellular adhesion molecule 1
CXCL: Chemokine (C-X-C motif) ligand
XAF1: XIAP-accociated factor 1
XIAP: X-linked inhibitor of apoptosis
PBAF: Polybromo-associated BAF
BAF: BRG1-associated factor
BRG1: Brahma-related gene 1
