# Supplementary material for: A genomic screen for angiosuppressor genes in the tumor endothelium identifies a multifaceted angiostatic role for bromodomain containing 7 (BRD7)
Source: Angiogenesis. 2017 Sep 26;20(4):641–54. doi: 10.1007/s10456-017-9576-3 (PMC5660147; doi:10.1007/s10456-017-9576-3)
Supplement: Supplementary file 1 — Supplementary material 1 (DOCX 36 kb) [file 10456_2017_9576_MOESM1_ESM.docx]

**Supplementary Material**

Figure S1 demonstrates primer validation. Figure S2 demonstrates the lack of involvement of DNA methylation and histone acetylation on BRD7 expression. Figure S3 shows that silencing RF24 and HMEC results in increased BRD7 expression. Figure S4 summarizes the effects of BRD7 overexpression and knockdown on angiogenic growth factors and receptors. Figure S5 shows the overlap of concordantly regulated genes after BRD7 overexpression and knockdown. Table S1 lists the 19 TEC suppressed transcripts. Table S2 lists primers sequences. Table S3 lists the overlaps of differentially regulated genes in the different datasets, such as visualized in figure S6. Table S4 lists the genes with >2-fold expression induction or suppression upon BRD7 knockdown after merging of data sets GSE22607, GSE65981 and GSE65981. Table S5 contains functional annotation clustering data by DAVID of genes upregulated (S5A, B) or downregulated (S5C) after BRD7 suppression.

**Supplemental Methods**

*Suppression subtractive hybridization and cDNA array screening*

EC were isolated from colorectal tumor tissues, patient-matched normal colon tissues and placenta tissues as previously described [1, 2]. Suppression subtractive hybridization (SSH) was done essentially as described previously [1], though here colon tumor EC (TEC) derived cDNA was used as driver cDNA and placenta EC (PLEC) and normal colon EC (NEC) were used as tester cDNA. Two separate subtractions were performed to create cDNA repertoires: (1) genes suppressed in TEC compared to NEC and (2) genes suppressed in TEC compared to PLEC. The resulting cDNA populations were cloned into a T/A cloning vector (Invitrogen) and transformed into *E.coli.* Inserts were amplified with universal M13 primers and spotted on nitrocellulose filters. Filters were separately hybridized with ^33^P-labeled cDNA (Amersham, Uppsala, Sweden) derived from TEC, NEC and PLEC. Spot intensities were analyzed using an Fx-phosphorimager (Bio-Rad) and QuantityOne software (Bio-Rad). Pair-wise comparisons between NEC, PLEC and TEC hybridizations were made to identify differentially expressed cDNAs after normalization for total signal. Positive controls and reference genes verified effective normalization. Inserts of bacterial clones corresponding to differential spots were sequenced and compared to the NCBI Nucleotide database using BLAST to reveal transcript identity.

*Promoter methylation analysis*

CpG island analysis of the region of 1000bp around the transcriptional start site of BRD7 was performed using EMBOSS CpG plot (<http://www.ebi.ac.uk/Tools/seqstats/emboss_cpgplot/>). DAC and TSA treatment, as well as methylation-specific PCR were performed as described previously [3].

*Immunohistochemistry*

Formalin-fixed, paraffin embedded tissue sections (5 μm) were dewaxed and incubated in 0.3%H_2_O_2_/methanol, followed by antigen retrieval in citric acid. Sections were blocked with PBS/1%BSA and rabbit anti-human BRD7 (Abcam; 1:50) was applied for 1 hr. Sections were washed in PBS and bound antibody was detected with biotinylated goat anti-rabbit IgG (Dako; 1:100). Staining was performed using Streptavidin-ABC complex (Dako) with DAB.

*Flow cytometry*

Cells were harvested with trypsin and subsequently fixed in ice-cold 70% EtOH for >2 hours on ice, or in 1% paraformaldehyde for 20 minutes at room temperature. Cells were incubated for 1 hour on ice in PBS/0.1%BSA with mouse anti-human ICAM1 (Monosan; 1:100). Primary antibodies were detected with FITC-conjugated streptavidin (Dako; 1:100), preceded by biotinylated rabbit anti-mouse IgG (Dako; 1:100). Cells were analyzed using a FACS Calibur (BD Biosciences) and data were analyzed using CellQuest software (BD Biosciences).

**Supplemental Results**

*Identification of angiosuppressor genes*

We previously described the identification of genes overexpressed in tumor EC (TEC) compared to normal EC (NEC) and placenta EC (PLEC), using suppression subtractive hybridization in combination with cDNA array screening [1]. In the present study, we queried for genes expressed in resting endothelium and specifically suppressed in TEC by reversing the subtraction procedure (Figure 1). The resulting cDNA repertoires were substantially biased towards repressed genes in TEC, as is evident from the skewed distribution of hybridization signal intensities (Figure 1B), confirming a successful subtraction. Included negative controls did not show differential expression, whereas positive controls from our previous studies demonstrated overexpression in TEC (Figure 1B, D). Putatively interesting candidate genes were defined as being downregulated by >3.5-fold in TEC as compared to both PLEC and NEC. Figure 1C shows that 170 transcripts were downregulated in TEC as compared to NEC, whereas 216 transcripts demonstrated suppression in TEC compared to PLEC. The corresponding clones of the 76 overlapping spots were subsequently sequenced to reveal the identity of the insert. Of these, 69 showed homology with known human genes or human expressed sequence tags by BLAST analysis against the NCBI databases; these sequences represented 19 different annotated genes (Figure 1D and Table S1).

*BRD7 expression is not regulated by epigenetic phenomena*

A CpG plot analysis revealed the presence of a CpG island in the BRD7 promoter region (Figure S3A), allowing putative expression regulation (gene silencing) by methylation of cytosine residues. However, using methylation-specific PCR of the indicated regions, we could neither detect promoter methylation in most colon tumor cell lines tested (with the exception of HCT116), nor in HUVEC (Figure S3B). The negative control (H2O) shows no bands, whereas the i*n vitro* methylated DNA (IVD) as positive control shows only the corresponding methylated DNA representing band. We previously demonstrated that treatment of EC with DNA methyltransferase (DNMT) and histone deacetylase (HDAC) inhibitors, 5-aza-2’-deoxycytidine (DAC) and trichostatin A (TSA), respectively, inhibits angiogenesis, associated with an increase in ICAM1 expression [4]. In contrast to the re-expression of ICAM1, no effects of DAC and TSA on the expression levels of BRD7 were observed (Figure S3C). Together, these data indicate that transcriptional silencing of BRD7 in activated EC does not involve promoter methylation or histone deacetylation.

**References**

1. van Beijnum JR, Dings RP, van der Linden E, et al (2006) Gene expression of tumor angiogenesis dissected: specific targeting of colon cancer angiogenic vasculature. Blood 108:2339–48. doi: 10.1182/blood-2006-02-004291

2. van Beijnum JR, Rousch M, Castermans K, et al (2008) Isolation of endothelial cells from fresh tissues. Nat Protoc 3:1085–91. doi: 10.1038/nprot.2008.71

3. Hellebrekers DMEI, Melotte V, Viré E, et al (2007) Identification of epigenetically silenced genes in tumor endothelial cells. Cancer Res 67:4138–4148. doi: 10.1158/0008-5472.CAN-06-3032

4. Hellebrekers DMEI, Castermans K, Viré E, et al (2006) Epigenetic regulation of tumor endothelial cell anergy: silencing of intercellular adhesion molecule-1 by histone modifications. Cancer Res 66:10770–7. doi: 10.1158/0008-5472.CAN-06-1609

**Supplemental Figure Legends**

*Figure S1: Primer design and validation*

A) Alignment of chicken (gg) and human (hs) BRD7 mRNA sequences with qPCR primer sequences highlighted. B) Validation of primer specificity. No amplification is observed with human primers (hs BRD7 #) on chicken (gg) cDNA or with chicken primers (gg BRD7 #) on human (hs) cDNA.

*Figure S2: BRD7 expression is not influenced by promoter methylation*

A) The BRD7 promoter (top panel) contains a CpG island (-417 to +437) covering the minimal promoter sequence (-293 to -168; grey arrow), the transcription start site (TSS), the 5’UTR and the first exon (black box) (top panel). Vertical black bars (middle panel) indicate individual CpGs within the CpG island predicted by EMBOSS CpGPlot (<http://www.ebi.ac.uk/Tools/emboss/cpgplot/index.html>) (lower panel). B) CpG methylation was measured by methylation-specific PCR using primer sets indicated by black arrowheads in (A). No methylation was detected in colon tumor cell lines (with the exception of HCT116 which shows a faint methylation band) nor in HUVEC (bottom panel), whereas negative (H2O) and positive (IVD) controls behaved as expected. C) Expression of BRD7 is not influenced by treatment of HUVEC with DAC, TSA or a combination of both, as measured by qPCR, in contrast to that of ICAM1 according to previously published results [3, 4].

*Figure S3: Increased BRD7 expression in silenced EC*

HMEC and RF24 were serum-starved (0.5% FCS instead of 10% FCS) for 48 hrs, and BRD7 expression was measured by qPCR. A clear increase of BRD7 mRNA is seen after silencing the cells.

*Figure S4: Profile of angiogenic factors and receptors after BRD7 overexpression and knockdown.*

A) mRNA expression after ectopic expression of full-length BRD7 (BRD7-FL) and bromodomain deletion mutant (BRD7-dBr). BRD7 protein expression after transfection was quantified by flow cytometry for flag-tag and shown on the right, indicating comparable transfection efficiencies. B) Effects of the different constructs vs untransfected cells on BRD7 protein expression (right; flag-tag detection), BRD7 mRNA expression (middle), and cell proliferation (right). C) mRNA expression after BRD7 knockdown using pooled siRNAs (siBRD7). D) mRNA expression after transfection with individual siRNAs (siBRD7_a and siBRD7_b), indicating comparable effects. Both siRNAs had no effect on HUVEC viability as shown on the right. From the three used siRNAs (see Materials and Methods section, SI04134088 showed least consistent results and was omitted from the analysis for suspected off-target effects.

*Figure S5: Venn diagrams of differentially expressed genes after BRD7 knockdown*

Overlap of differentially regulated (Log2FC > 1 or <-1) genes in the indicated gene expression data sets. Up/down regulated reflects ratios of shRNA/Ctrl in GSE20076 and GSE22607 and Ctrl/pDNA in GSE65981 to have similar directionality. Gene expression data of BJ1 fibroblast cells transfected with shRNA targeting BRD7 (GSE22607 and GSE20076; BRD7 knockdown) and data of HEK293 fibroblasts transfected with BRD7 pDNA (GSE65981; BRD7 induction) were evaluated for overlapping genes. Venn diagrams of up- and downregulated genes following BRD7 suppression are indicated. The corresponding gene lists are presented in Table S3.
