# Supplementary material for: A genomic screen for angiosuppressor genes in the tumor endothelium identifies a multifaceted angiostatic role for bromodomain containing 7 (BRD7)
Source: Angiogenesis. 2017 Sep 26;20(4):641–54. doi: 10.1007/s10456-017-9576-3 (PMC5660147; doi:10.1007/s10456-017-9576-3)
Supplement: Supplementary file 2 — Supplementary material 2 (PDF 84 kb) [file 10456_2017_9576_MOESM2_ESM.pdf]

A

```
hsBRD7  GGATAAAAAGAAGCGAGATCGAGACCGGGTGGAGAATGAGGCAGAAAAAGATCTCCAGTG 480
ggBRD7  GGATAAGAGGAAACGCGATCGAGAACACCCAGACAGTGAGGGAGAGCAGGAACTGAGATG 405
        ****** * *** ** ******** *     ** * ***** ***  * ** **    **

hsBRD7  TCACGCCCCTGTGAGATTAGACTTGCCTCCTGAGAAGCCTCTCACAAGCTCTTTAGCCAA 540
ggBRD7  TCAGACCCCTATCAGATTGGAATTGTCACCAGAGAAACCATTGACGAGTTCTTTATCAAA 465
        ***  ***** * ***** ** *** * ** ***** **  * ** ** ****** * **
```

B

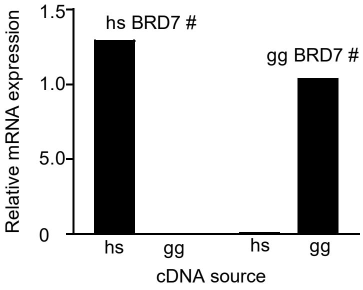

Fig. S1

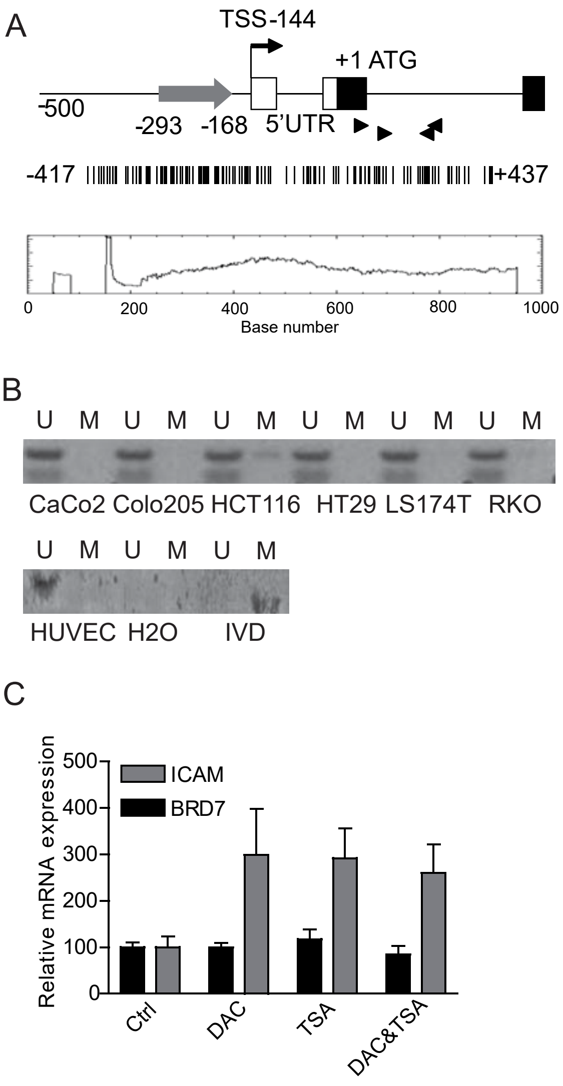

A
TSS-144
+1 ATG
-500
-293
-168
5'UTR
-417
+437
0
200
400
600
800
1000
Base number
B
U M U M U M U M U M U M
CaCo2 Colo205 HCT116 HT29 LS174T RKO
U M U M U M
HUVEC H2O IVD
C
Relative mRNA expression
500
400
300
200
100
0
ICAM
BRD7
Ctrl
DAC
TSA
DAC&TSA

Fig.S2

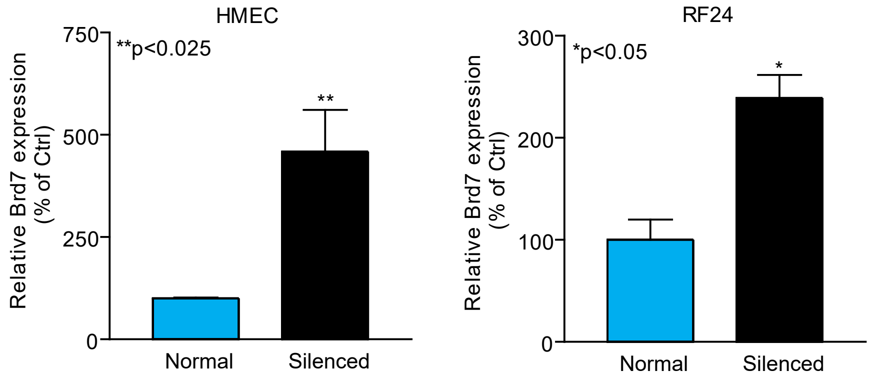
HMEC
**p<0.025
750
500
250
0
Relative Brd7 expression (% of Ctrl)
**
Normal
Silenced
RF24
*p<0.05
300
200
100
0
Relative Brd7 expression (% of Ctrl)
*
Normal
Silenced

Fig.S3

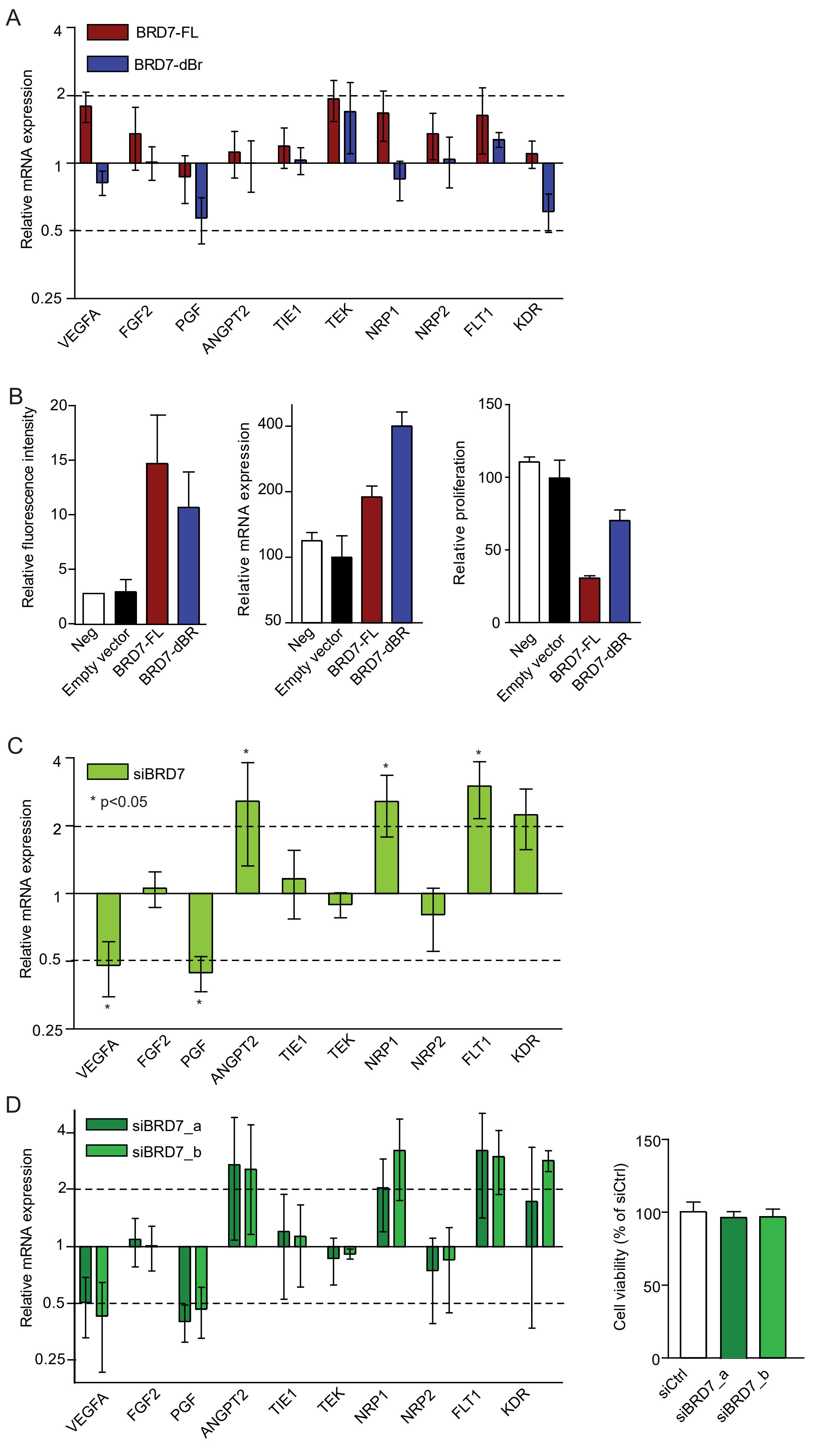
A
BRD7-FL
BRD7-dBr
Relative mRNA expression
4
2
1
0.5
0.25
VEGFA
FGF2
PGF
ANGPT2
TIE1
TEK
NRP1
NRP2
FLT1
KDR
B
Relative fluorescence intensity
20
15
10
5
0
Neg
Empty vector
BRD7-FL
BRD7-dBR
Relative mRNA expression
400
200
100
50
Relative proliferation
150
100
50
0
C
siBRD7
* p<0.05
Relative mRNA expression
D
siBRD7_a
siBRD7_b
Relative mRNA expression
Cell viability (% of siCtrl)
siCtrl
siBRD7_a
siBRD7_b

Fig.S4

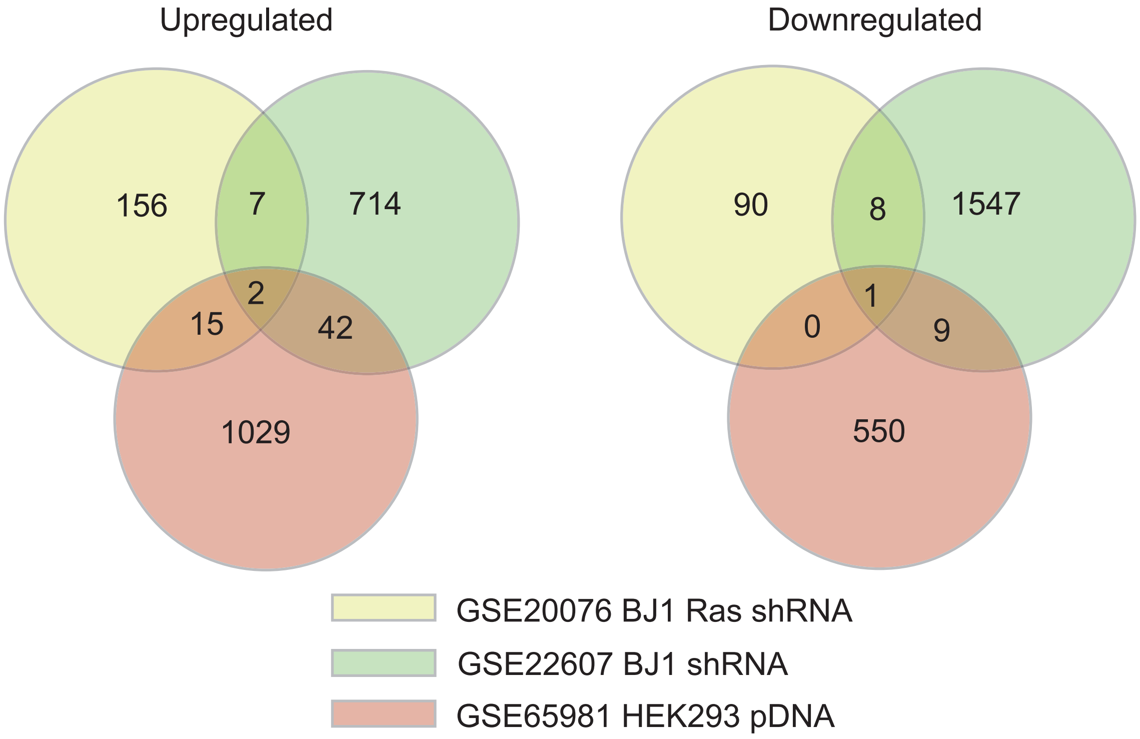

Upregulated
156
7
714
2
15
42
1029
Downregulated
90
8
1547
1
0
9
550
GSE20076 BJ1 Ras shRNA
GSE22607 BJ1 shRNA
GSE65981 HEK293 pDNA

Figure S5
