# Supplementary material for: A genomic screen for angiosuppressor genes in the tumor endothelium identifies a multifaceted angiostatic role for bromodomain containing 7 (BRD7)
Source: Angiogenesis. 2017 Sep 26;20(4):641–54. doi: 10.1007/s10456-017-9576-3 (PMC5660147; doi:10.1007/s10456-017-9576-3)
Supplement: Supplementary file 3 — Supplementary material 3 (PDF 61 kb) [file 10456_2017_9576_MOESM3_ESM.pdf]

Supplementary Table 1: Genes suppressed in tumor endothelial cells

Name	Accession No	Description	Average Log10 normalized intensity TEC	NEC	PLEC
AFF4	NM_014423.3	Homo sapiens AF4/FMR2 family, member 4 (AFF4), mRNA	4.629	5.199	5.364
API5	NM_006595.2	Homo sapiens apoptosis inhibitor 5 (API5), mRNA	5.237	6.007	6.230
BRD7	NM_013263.2	Homo sapiens bromodomain containing 7 (BRD7), mRNA	4.762	5.338	5.528
DAD1	NM_001344.1	Homo sapiens defender against cell death 1 (DAD1), mRNA	4.270	4.875	6.449
EEFG1	NM_001404.1	Homo sapiens eukaryotic translation elongation factor 1 gamma (EEF1G), mRNA	5.460	6.112	6.344
GALNT1	NM_020474.2	Homo sapiens UDP-N-acetyl-alpha-D-galactosamine:polypeptide N-acetylgalactosaminyltransferase 1 (GalNAc-T1)	4.984	5.530	5.817
GFPT2	NM_005110.2	Homo sapiens glutamine-fructose-6-phosphate transaminase 2 (GFPT2)	5.223	5.816	5.998
GIT2	NM_057169.2	Homo sapiens G protein-coupled receptor kinase interactor 2 (GIT2), transcript variant 1	4.891	5.560	5.884
GUCY2C	NM_004963.1	Homo sapiens guanylate cyclase 2C (heat stable enterotoxin receptor) (GUCY2C), mRNA	5.200	6.089	6.097
HNRPDL	NM_031372.2	Homo sapiens heterogeneous nuclear ribonucleoprotein D-like (HNRPDL), transcript variant 2, mRNA	5.739	6.904	6.939
HSD17B6	NM_003725.2	Homo sapiens hydroxysteroid (17-beta) dehydrogenase 6 (HSD17B6), mRNA	5.022	5.710	5.871
IGJ	NM_144646.2	Homo sapiens immunoglobulin J polypeptide, linker protein for immunoglobulin alpha and mu polypeptides (IGJ), mRNA	5.071	5.818	5.741
MBNL2	NM_144778.2	Homo sapiens muscleblind-like 2 (Drosophila) (MBNL2), transcript variant 1&3, mRNA	4.905	5.525	5.557
MFAP5	NM_003480.2	Homo sapiens microfibrillar associated protein 5 (MFAP5), mRNA	5.163	5.737	5.778
OGN	NM_033014.2	Homo sapiens osteoglycin (osteoinductive factor, mimecan) (OGN), 3 transcript variants, here variant 1, mRNA	4.918	5.506	5.750
RPL34	NM_000995.3	Homo sapiens ribosomal protein L34 (RPL34), transcript variants 1&2, mRNA, here var1	5.511	6.803	6.925
SNAP29	NM_004782.2	Homo sapiens synaptosomal-associated protein, 29kDa (SNAP29), mRNA	5.028	5.673	5.719
TACSTD1	NM_002354.1	Homo sapiens tumor-associated calcium signal transducer 1 (TACSTD1), mRNA	4.887	5.572	5.642
TUFT1	NM_020127.1	Homo sapiens tuftelin 1 (TUFT1), mRNA	4.869	5.537	5.793
