# Supplementary material for: A genomic screen for angiosuppressor genes in the tumor endothelium identifies a multifaceted angiostatic role for bromodomain containing 7 (BRD7)
Source: Angiogenesis. 2017 Sep 26;20(4):641–54. doi: 10.1007/s10456-017-9576-3 (PMC5660147; doi:10.1007/s10456-017-9576-3)
Supplement: Supplementary file 4 — Supplementary material 4 (PDF 70 kb) [file 10456_2017_9576_MOESM4_ESM.pdf]

Supplementary Table 2: Primer sequences

Name *	Fw	Rev
hsBRD7	TCAAGACAGGCTCATAGCGCT	TTCATTCTGGGCTTCCTGGAG
hsBRD7	TGAAGGCCATACTAGGACACTT	CGCTATGAGCCTGTCTTGAGTA
hsBRD7	AGCGAGATCGAGACCGGGT	TTGGCTAAAGAGCTTGTGAGA
ggBRD7	ACGCGATCGAGAACACCCA	TGATAAAGAACTCGTCAATGGT
hsPPIA	CTCGAATAAGTTTGACTTGTGTTT	CTAGGCATGGGAGGGAACA
ggPPIA	AAGGAGGGGATGAACGTG	AGCTGCCCGCAGTTGGA
hsACTB	CATTCCAAATATGAGATGCATT	CCTGTGTGGACTTGGGAGAG
ggACTB	AGACAGCTACGTTGGTGATGAA	TGCTCCTCAGGGGCTACTCT
hsB2M	TCCATCCGACATTGAAGTTG	CGGCAGGCATACTCATCTT
ggB2M	ACCCACCCAGGATCACCA	TGTAGACGGCTTCGCTGC
ICAM1	GGCCGGCCAGCTTATACAC	TAGACACTTGAGCTCGGGCA
TNF	GGCGTGGAGCTGAGAGAT	TGGTAGGAGACGGCGATG
CXCL1	AGGAGGAAGCTCACTGGTGGC	AGCGATGCTCAAACACATTAGGC
CXCL6	AGTTTCCTGCCAGTCGGG	TCAAGAGAGGGTTCGCGG
NOTCH1	TCAGACGGTGCCCTCATGG	ACTGCCGGTGGTCTGTCTGG
VEGFA	AAGGAGGAGGGCAGAATCAT	CCAGGCCCTCGTCATTG
FGF2	CCCGACGGCCGAGTTGAC	CACATTTAGAAGCCAGTAATCT
PGF	TGCAGCTCCTAAAGATCCGT	GGGAACAGCATCGCCGCA
ANGPT2	TGCCACGGTGAATAATTCAG	TTCTTCTTTAGCAACAGTGGG
TIE1	CCCCGCTGGTCTCGTTCTC	CACAATGGTCGACCAGTCC
TEK	TTGAAGTGGAGAGAAGGTCTG	GTTGACTCTAGCTCGGACCAC
NRP1	CCCGAGAGAGCCACTCATG	GTCATCACATTCATCCACCAA
NRP2	CAATTGCAACTTCGATTTCCTC	CCGGTCGTTTGGGCTGGA
FLT1	CCAGCAGCGAAAGCTTTGCG	CTCCTTGTAGAAACCGTCAG
KDR	ATGACATTTTGATCATGGAGC	CCCAGATGCCGTGCATGAG

* Presets hs and gg denote species (human and chicken) selective primers, designed to amplify indicated transcript from complex cDNA samples
