# Supplementary material for: A genomic screen for angiosuppressor genes in the tumor endothelium identifies a multifaceted angiostatic role for bromodomain containing 7 (BRD7)
Source: Angiogenesis. 2017 Sep 26;20(4):641–54. doi: 10.1007/s10456-017-9576-3 (PMC5660147; doi:10.1007/s10456-017-9576-3)
Supplement: Supplementary file 5 — Supplementary material 5 (PDF 56 kb) [file 10456_2017_9576_MOESM5_ESM.pdf]

Supplementary Table 3: Overlap expression data sets

3a Inversely related with BRD7 expression

GSE22607 BJ & GSE65981 HEK293	GSE20076 BJ Ras & GSE65981 HEK293	GSE22607 BJ & GSE20076 BJ Ras	Overlap All
ABI3BP	BST2	TUBB2B	CXCL1
AIF1L	C1QTNF6	STK32B	CXCL6
AMPH	COL1A1	HES2	
B4GALNT4	COL6A2	FAM84B	
BIRC3	DDIT4	ADA	
C1orf21	DENND2A	LOC375295	
C3orf23	EFEMP1	SYT15	
C8orf46	IL8		
CCDC148	PARP12		
CD83	PTX3		
CLDN15	SCG2		
CLEC2D	SOCS2		
COX6B2	THBS2		
CXCL2	TRIM47		
DNER	ZNF185		
DNM3			
DPY19L2			
DTD1			
ERBB3			
ESRRB			
FLJ23152			
FLT4			
FRAS1			
GALC			
GFRA1			
GPR160			
GUCY1B3			
HSPA14			
IGSF3			
INHBA			
KCNN1			
KIAA1109			
NOTCH1			
P4HTM			
PPP2R2C			
RAB3D			
SC5DL			
SLC1A3			
SLC7A11			
SOX11			
STRBP			
ZNF441			
