# Supplementary material for: A genomic screen for angiosuppressor genes in the tumor endothelium identifies a multifaceted angiostatic role for bromodomain containing 7 (BRD7)
Source: Angiogenesis. 2017 Sep 26;20(4):641–54. doi: 10.1007/s10456-017-9576-3 (PMC5660147; doi:10.1007/s10456-017-9576-3)
Supplement: Supplementary file 6 — Supplementary material 6 (PDF 55 kb) [file 10456_2017_9576_MOESM6_ESM.pdf]

Supplementary Table 3: Overlap expression data sets

3b Concordantly related with BRD7 expression

GSE22607 BJ & GSE65981 HEK293	GSE20076 BJ Ras & GSE65981 HEK293	GSE22607 BJ & GSE20076 BJ Ras	Overlap All
TNFRSF21		FNDC4	BRD7
CYP7B1		ADAMTS1	
ELFN2		GAL	
DPYSL5		PRSS3	
UNC5C		TAGLN3	
TAF7L		IGFBP2	
DOCK8		ACPP	
RFPL4B		CCRL1	
APOC1			
